# Kombucha Prevents Indomethacin‐Induced Enteric Damage in Wistar Rat by Enhancing Epithelial Gut Barrier and Modulating Gut Microbiota

**DOI:** 10.1002/fsn3.70804

**Published:** 2025-08-16

**Authors:** Atziri A. Varela‐Mendoza, Ma. Magdalena Martínez‐Flores, Melanie G. Paz‐Jiménez, Fernanda García‐Acevedo, Laura E. Córdova‐Dávalos, Tonatiuh Barrios‐García, Ma. Consolación Martínez‐Saldaña, Valeria Salinas‐Guardado, Mariela Jiménez, Eva Salinas, Daniel Cervantes‐García

**Affiliations:** ^1^ Department of Microbiology, Basic Science Center Autonomous University of Aguascalientes Aguascalientes Mexico; ^2^ Department of Morphology, Basic Science Center Autonomous University of Aguascalientes Aguascalientes Mexico; ^3^ Department of Nutrition, Health Science Center Autonomous University of Aguascalientes Aguascalientes Mexico; ^4^ Secretariat of Science, Humanities, Technologies and Innovation Mexico City Mexico

**Keywords:** gut inflammation, kombucha, microbiota, NSAIDs, polyphenols, tight junctions

## Abstract

Kombucha is a fermented beverage with high contents of antioxidants and probiotics. Enteric damage due to uncontrolled consumption of non‐steroidal anti‐inflammatory drugs (NSAIDs) represents a significant cause of morbidity and mortality. This study aimed to evaluate the preventive effects of kombucha against indomethacin‐induced enteric damage. Wistar rats were divided into control (Ctrl), supplemented with kombucha (Komb), indomethacin‐induced enteric damage (Indo), and indomethacin‐induced enteric damage and supplemented with kombucha (Indo‐Komb) groups. We analyzed ulcer index, histological changes, gene expression of intestinal barrier function genes, and microbiota phyla in cecal content. Kombucha supplementation reduced ulcer index and intestinal inflammation, which improved survival (*p* = 0.0291), maintained body weight, (*p* < 0.001) and avoided the drop in levels of hemoglobin, hematocrit, and erythrocytes (*p* < 0.001). Histopathological analyses of intestines revealed impaired integrity of villi with inflammatory infiltrates, which were significantly prevented with kombucha supplementation (*p* = 0.0024 and *p* = 0.0017, respectively). Rats that received kombucha upregulated the expression of tight junction genes *Muc2*, *Cldn1*, and *Ocln* (*p* = 0.027, *p* = 0.024, *p* < 0.001, respectively). The upregulation of the oxidative and inflammatory markers *Nos2*, *Mpo*, and *Tnf* was prevented by kombucha intake (*p* < 0.001). Additionally, kombucha ameliorated the dysbiosis in indomethacin‐induced enteric damaged rats by reduction of *Proteobacteria* (*p* < 0.001) while increasing *Firmicutes* abundance. This study demonstrated that kombucha consumption is a viable strategy to delay NSAID‐induced enteric damage through enhancement of intestinal barrier integrity and prevention of dysbiosis.

## Graphical abstract

Indomethacin‐induced enteric damage is notoriusly reduced by kombucha supplementation. Kombucha reduced inflammation and prevents epithelial damage.

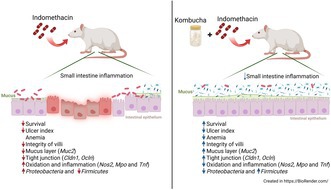

## 1 Introduction

Kombucha is a fermented beverage with various healing properties (Andreson et al. 2022). Traditionally, it is prepared by fermentation of an infusion of sweetened black or green tea with a symbiotic culture of bacteria and yeasts (SCOBY), resulting in a beverage with acidic and carbonated organoleptic properties (Júnior et al. 2022). SCOBY is a complex symbiotic consortium composed of different genera of yeasts and a broad bacterial community, mainly acetic acid and lactic acid bacteria (Villarreal‐Soto et al. 2018). Kombucha fermentation releases a huge amount of postbiotics, such as short chain fatty acids (SCFA, mainly including acetate, propionate, and butyrate), organic acids, polyphenols, vitamins, amino acids, and biogenic amines (Martínez Leal et al. 2018; Zhou et al. 2022).

Non‐Steroidal Anti‐inflammatory Drugs (NSAIDs) are first‐line treatment options for relieving acute pain in patients, due to their important analgesic, antipyretic, and anti‐inflammatory activities (Ungprasert et al. 2016). Since NSAIDs are easily accessible and often unsupervised by a health care provider, their use has been broadly extended, and it is estimated that thousands of tons of NSAIDs are consumed every year worldwide (Brennan et al. 2021; Lin et al. 2023). However, NSAIDs are frequently linked to several gastrointestinal conditions, such as ulceration, perforation, obstruction, and hemorrhage (Ong et al. 2007). NSAIDs‐induced enteric damage occurs through the inhibition of cyclooxygenases (COXs), leading to a blockade of the synthesis of prostaglandins (PGs), molecules that are essential for the mucosal defense system in the small bowel (Watanabe et al. 2020). Intestinal damage is also mediated by a reduction in mucus secretion, enterocyte survival and differentiation, and the amount of tight junction proteins, which is accompanied by dysbiosis. The increase in mucosal permeability promotes enterobacterial and acid juice translocation and the subsequent intestinal inflammation and oxidative stress, which are characterized by neutrophils influx and upregulation of inducible nitric oxide synthase (NOS2) (Miyoshi et al. 2017; Shi et al. 2014; Takeuchi and Satoh 2015; Zhu et al. 2023). Therefore, new therapeutic strategies aimed at strengthening the gut antioxidant response, improving the intestinal barrier, and maintaining equilibrated microbiota may lead to the prevention of enteric damage by NSAIDs.

Antioxidant compounds, such as hemin, glycomacropeptide, quercetin, resveratrol, rutin, and epigallocatechin gallate, have shown to be effective in preventing the indomethacin‐induced intestinal injury (Carrasco‐Pozo et al. 2012; Cervantes‐García et al. 2020; Yoriki et al. 2013). Thus, it is feasible to consider kombucha as a promising functional beverage to prevent intestinal disorders in which homeostasis is compromised. This study aimed to evaluate the prophylactic effects of kombucha supplementation on indomethacin‐induced enteric damage in Wistar rats.

## 2 Materials and Methods

### 2.1 Experimental Animals and Ethical Statements

All experimental protocols were approved by the Research Ethics Committee of the Autonomous University of Aguascalientes (CEADI‐UAA/02/2025) and were carried out in accordance with the institutional guidelines for experimental animal care and the national regulatory norm (NOM‐062‐Z00‐1999). Twenty‐five male Wistar rats (100–140 g) were obtained from the Central Animal Care Laboratory of the Autonomous University of Aguascalientes. Animals were maintained on a light/dark cycle (12 h:12 h) and controlled temperature of 22°C ± 2°C with *ad libitum* access to pellet diet (Rodent Laboratory Chow 5001; Purina, Mexico) and tap water. Animals were housed in polycarbonate cages (5 rats per cage) for a week for acclimatization.

### 2.2 Indomethacin‐Induced Damage Model and Sample Collection

**FIGURE 1:**
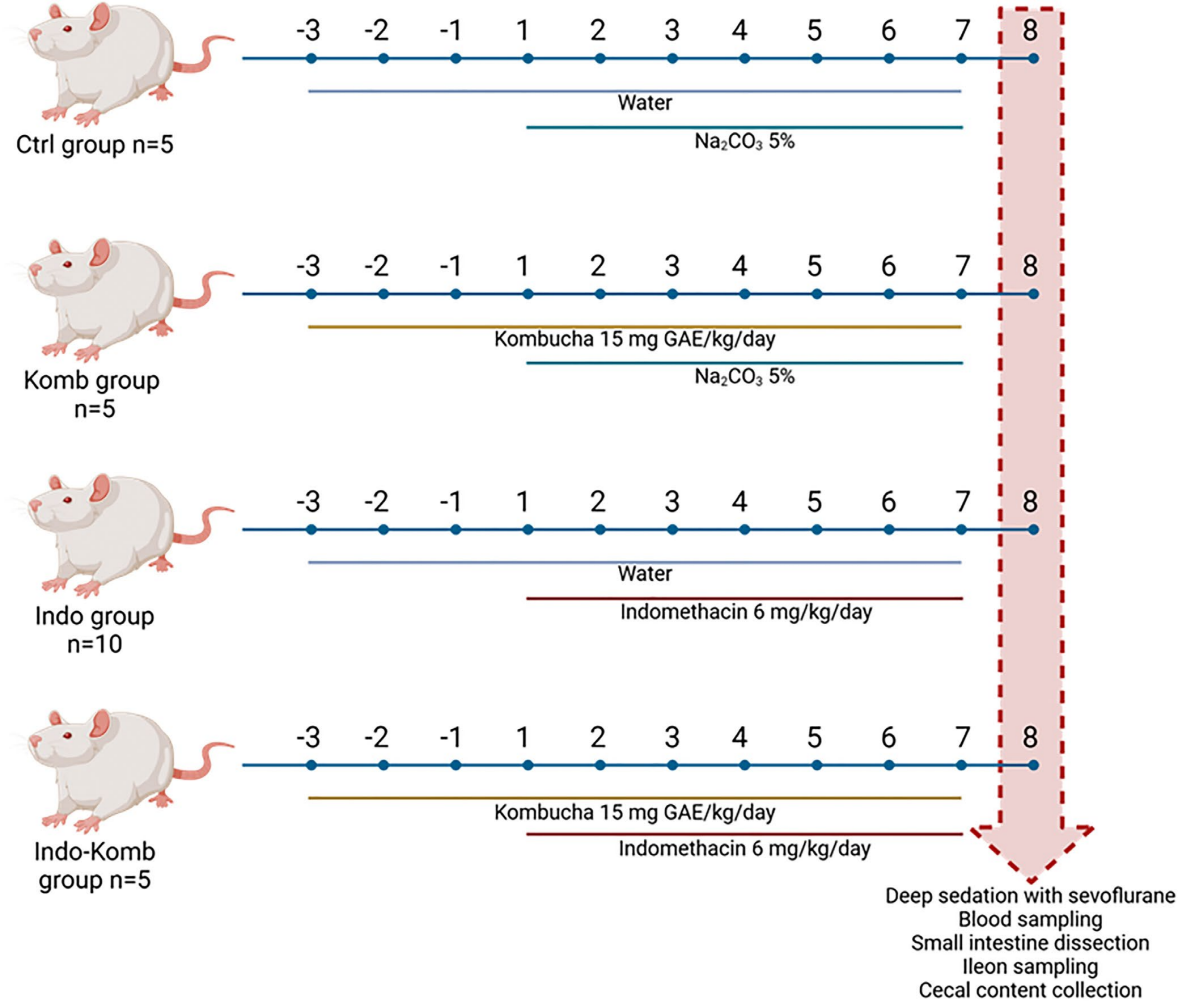
Schematic representation of experimental design and sampling. Rats of the groups Indo and Indo‐Komb were administered by esophageal gavage with indomethacin 6 mg/kg/day for 7 days (Days 1 to 7), while rats of the groups Ctrl and Komb received sodium carbonate 5% solution. Kombucha was orally dosed at 15 mg GAE/kg from Day −3 to Day 7. On Day 8, rats were deeply sedated to obtain blood samples, and small intestines were dissected for macroscopic analyses of ulcers. Tissue samples of ileum were obtained for histopathological and gene expression analyses. Cecal content was collected to evaluate microbial phyla. Created with BioRender.com.

Animals were randomly assigned to the following experimental groups: (i) control group (Ctrl, *n* = 5), (ii) kombucha administered group (Komb, *n* = 5), (iii) indomethacin‐induced damage group (Indo, *n* = 10; to ensure *n* ≥ 5 on Day 8), and (iv) indomethacin‐induced damage and kombucha administered group (Indo‐Komb, *n* = 5). Kombucha was orally administered with an esophageal gavage in animals in the Komb and Indo‐Komb groups for 10 days (−3 to 7 day), considering a total phenolic intake of 15 mg/kg of body weight (Banerjee et al. 2010); on the same days, Ctrl and Indo rats received 1 mL of tap water. Indomethacin (Sigma‐Aldrich, St. Louis, MO, USA) dissolved in Na_2_CO_3_ 5% was orally administered with an esophageal gavage at a daily dose of 6 mg/kg for 7 days (starting 3 days after kombucha administration, i.e., 1 to 7 days) to Indo and Indo‐Komb animals, while Ctrl and Komb rats received 1 mL 5% Na_2_CO_3_ as vehicle (Figure 1). Rat weight and mortality were registered during the time of indomethacin administration. On Day 8, animals were sedated with inhaled sevoflurane (PiSA, CDMX, Mexico), and blood was obtained from the abdominal aorta. The small intestine was dissected, washed with phosphate‐buffered saline (PBS, pH 7.4), and weighed. Two segments of 1 cm from the ileum were obtained; one was fixed in 10% neutral formalin for histological analysis, and another was immersed in DNA/RNA Shield (Zymo Research, Irvine, CA, USA) and stored at −80°C until use for gene expression studies. Macroscopical analyses were carried out on the remaining small intestine. Cecal content was recovered for microbiota analysis.

### 2.3 Kombucha Preparation

Kombucha was prepared with a base of green tea as previously described (Costa et al. 2022). Briefly, green tea infusion was prepared with 12 g of tea leaves and 50 g of common sugar per liter of drinking water at 75°C for 3 min. Once the beverage had cooled to room temperature, tea leaves were removed by filtration (Whatman filter No. 1), and a SCOBY (3% w/v, acquired in a local market) and previously prepared kombucha (10% v/v) were added and maintained in a glass flask at 25°C for 10 days. Then, the SCOBY was manually removed, and the beverage was stored at 4°C for up to 1 week to be administered to animals.

### 2.4 Total Phenolic Content Determination in Kombucha

The content of phenolic compounds was determined using the Folin–Ciocalteu reagent (Sigma Aldrich) with gallic acid (Sigma Aldrich) as standard. Briefly, a volume of 180 μL of kombucha, or standard solution of gallic acid, was mixed with 20 μL of Folin–Ciocalteu reagent and then incubated in the dark for 10 min. Next, 50 μL of sodium carbonate 1% solution was added and incubated for 20 min in the dark. Absorbance was measured at 750 nm in a microplate reader iMark (Bio Rad, Hercules, CA, USA), and data was obtained as Gallic Acid Equivalents (GAE).

### 2.5 Macroscopic Evaluation of the Small Intestines

The small intestines were longitudinally opened along the antimesenteric border, washed with PBS, and fixed in 10% neutral formalin for 24 h. Then, the intestines were rinsed with 70% ethanol for 30 min. Ulcerations were quantified and measured using a digital caliper on the inner side of the intestine. The ulcer index was calculated as the total ulcerated area (mm^2^) per intestine.

### 2.6 Hematic Biometry Test

Hematological parameters were determined in EDTA‐anticoagulated blood in an automated hematologic analyzer Orpheé Mythic 18 (Diamond Diagnostics, Holliston, MA, USA).

### 2.7 Histological Evaluation of the Small Intestines

Formalin‐fixed small intestine tissues were paraffin‐embedded, cut into 4–5 μm sections, and mounted on silanized glass slides. Slides were deparaffinized, rehydrated, and stained with hematoxylin and eosin; micrographs were obtained with a DM750 microscope (Leica, Wetzlar, GER) equipped with a digital camera ICC50W (Leica). Images were analyzed with a validated scoring system (Lázár et al. 2019) by two examiners who were unaware of the animal treatment.

### 2.8 Quantitative PCR for Analysis of Barrier Function and Inflammation Gene Expression

Total RNA was extracted from stored tissues using the GeneJET RNA Purification Kit (Thermo Scientific, Waltham, MA, USA) according to the manufacturer's instructions. The evaluation of RNA was performed using the NanoDrop2000 (Thermo Scientific), considering an A260/A280 ratio > 1.8 acceptable for RNA quality. mRNA was reverse transcribed to cDNA with the First Strand cDNA Synthesis Kit (Thermo Scientific). Quantitative real‐time PCR was performed using the Maxima SYBR Green/ROX qPCR Master Mix 2× (Thermo Scientific) in a StepOne Real‐Time PCR System (Thermo Scientific). The primers used are listed in Table 1. Gene expression levels were normalized with the housekeeping gene *Actb* and using the 2^−ΔΔ*Ct*^ method (Schmittgen and Livak 2008).

**TABLE 1:** Primer sequences used for gene expression analyses.

Gene	Oligonucleotide	Accession number
*Muc2*	Fw: GTATGTGCTCGCCTGTATGC	NM_022174.1
	Rv: TGACCTCCAGATGTGAGCAG	
*Cldn1*	Fw: AACCTCTTACCCAACACCACG	NM_031699.2
	Rv: GCCAAGACCCTCATAGCCAT	
*Ocln*	Fw: AGGACAGACCCAGACCACTA	NM_031329.2
	Rv: ACTCTTCGCTCTCCTCTCTG	
*Nos2*	Fw: GATGTGCTGCCTCTGGTCCT	NM_012611.4
	Rv: ACTCCAATCTCGGTGCCCAT	
*Mpo*	Fw: TCACATACCGAGACTACCTGCC	NM_001401812.1
	Rv: AAGGGTTGGATGAGGGTGTG	
*Tnf*	Fw: GCCTCAGCCTCTTCTCATTCCT	NM_012675.3
	Rv: CGCTTGGTGGTTTGCTACGA	
*Actb*	Fw: GTCGTACCACTGGCATTGTG	NM_031144.3
	Rv: GCTGTGGTGGTGAAGCTGTA	

### 2.9 Microbiota Evaluation in Cecal Content

Cecal content samples (100–150 mg) were employed for genomic DNA extraction using the E.Z.N.A. Stool DNA kit (Omega Bio‐Tek, Norcross, GA, USA) following the manufacturer's protocol. DNA quality and quantity were assessed with a NanoDrop 2000 (Thermo Scientific). Bacterial phyla were quantified by amplification of the *16S* rRNA gene with specific oligonucleotides listed in Table 2. qPCR was performed with the Maxima SYBR Green/ROX qPCR Master Mix 2× (Thermo Scientific) in a StepOne Real‐Time PCR system (Thermo Scientific). For absolute quantification, amplicons for each phylum were cloned into the InsTAclone PCR Cloning kit (Thermo Scientific) to generate the plasmids pTZ57R‐Firmicutes, pTZ57R‐Bacteroidetes, pTZ57R‐Proteobacteria, and pTZ57R‐Actinobacteria; then, serial 10‐fold dilutions of plasmids with amplicon cloned were used as templates to establish a standard curve for the *16S* rRNA gene of each phylum. Results are presented as a ratio of each experimental group phylum to its respective control group.

**TABLE 2:** Primer sequences for evaluation of cecal bacterial phyla.

Microorganisms	Oligonucleotide	References
*Firmicutes*	Fw: GGAGYATGTGGTTTAATTCGAAGCA	Rizzardi et al. (2021)
	Rv: AGCTGACGACAACCATGCAC	
*Bacteroidetes*	Fw: GGARCATGTGGTTTAATTCGATGAT	Rizzardi et al. (2021)
	Rv: AGCTGACGACAACCATGCAG	
*Proteobacteria*	Fw: CATGACGTTACCCGCAGAAGAAG	Murri et al. (2013)
	Rv: CTCTACGAGACTCAAGCTTGC	
*Actinobacteria*	Fw: GADACYGCCGGGGTYAACT	Pfeiffer et al. (2014)
	Rv: TCWGCGATTACTAGCGAC	

### 2.10 Statistical Analysis

All data were analyzed using the GraphPad Prism 8.0 software (GraphPad Software Inc., La Jolla, CA, USA) and presented as the mean ± standard error of the mean (SEM). Lower and upper 95% confidence intervals (CI) of results were included. Kaplan–Meier survival curves were compared with a log‐rank test. Comparisons among groups were performed using the one‐way analysis of variance (ANOVA) followed by a Bonferroni's test. Statistical significance was considered when *p* < 0.05.

## 3 Results

### 3.1 Effect of Kombucha Treatment on Weight Loss and Survival in Indomethacin‐Administered Animals

**FIGURE 2:**
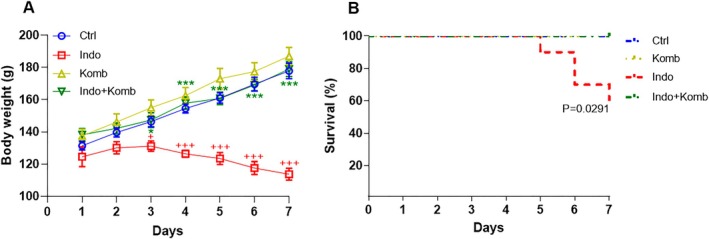
Effect of kombucha on body weight (A) and survival rate (B) in rats with indomethacin‐induced enteric damage. ^+^ *p* < 0.05 and ^+++^ *p* < 0.001 vs. Ctrl; *p < 0.05 and ***p < 0.001 vs. Indo.

The total content in polyphenols of kombucha was 1.85 mg GAE/mL. This datum allowed us to determine the volume of kombucha we had to administer to rats for 15 mg GAE intake/animal. During the time of induction of intestinal damage with indomethacin, the rats showed evident loss of body weight (Figure 2A). The Indo group showed significantly reduced weight since Day 3 of indomethacin administration (CI 123.9 to 138.3 g; *p* = 0.043), which continued to decrease significantly until Day 7 (CI 104.1 to 123.2 g; *p* < 0.001) when compared to the Ctrl group (CI 139.0 to 153.8 and 167.0 to 188.8 g; *p* < 0.001). Both groups administered with kombucha resulted in a similar body weight gain rate to that of the Ctrl group. The Indo‐Komb group showed significantly reduced body weight loss since Day 3 (CI 137.1 to 157.7 g; *p* = 0.0267), which was constantly maintained until Day 7 (CI 168.4 to 189.6 g; *p* < 0.001) when compared to the Indo group. As shown in Figure 2B, no death occurred in Ctrl and Komb groups over 7 days of vehicle or kombucha administration. Contrastingly, one animal from the Indo group died on Day 5 (90% survival, 9/10), 2 animals on Day 6 (70% survival, 7/10), and one more animal on Day 7 (60% survival, 6/10). This survival rate was even lower than that previously observed (Cervantes‐García et al. 2020); therefore, we analyzed five animals at the end of the protocol for the Indo group. The survival rate in the Indo‐Komb group was significantly improved (100%) compared to that of the Indo group (*p* = 0.0291).

### 3.2 Effect of Kombucha Treatment on Intestinal Weight, Length, and Edema in Indomethacin‐Administered Animals

**FIGURE 3:**
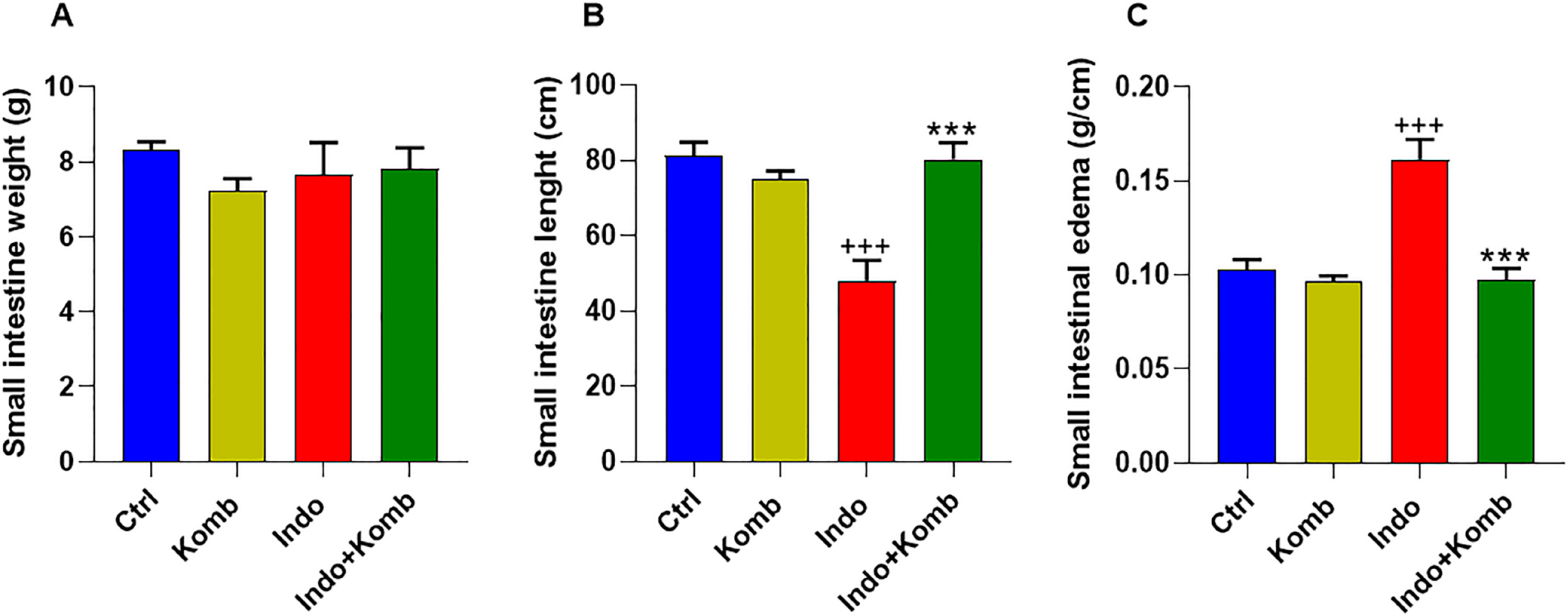
Kombucha supplementation alleviates gut inflammation in rats with indomethacin‐induced enteric damage. Small intestine weight (A), length (B), and edema (C) from experimental animals. ^+++^ *p* < 0.001 vs. Ctrl; ***p < 0.001 vs. Indo.

Administration of kombucha and/or indomethacin had no effect on the weight of small intestines (Figure 3A). However, after 7 days of indomethacin administration, the intestinal length in Indo group animals was significantly reduced by 41.03% the intestinal length as compared to that of the Ctrl group (CI 32.82 to 63.18 cm vs. 71.64 to 91.16 cm; *p* < 0.001). Remarkably, Indo‐Komb animals showed an intestinal length comparable to that of the Ctrl group (CI 68.41 to 92.39 cm), but was significantly increased by 0.67‐fold than the Indo group (*p* < 0.001) (Figure 3B). Moreover, the weight‐length ratio, a parameter used to estimate gut edema and inflammation (Nazari‐Khanamiri et al. 2023), significantly increased by 0.56‐fold in the Indo group compared with the Ctrl group (CI 0.132 to 0.191 vs. 0.088 to 0.118; *p* < 0.001). Beneficially, kombucha administration avoided small intestinal edema, as weight‐length ratio in the Indo‐Komb group was significantly 39.61% lower (CI 0.081 to 0.114) than that of the Indo group (*p* < 0.001) (Figure 3C).

### 3.3 Effect of Kombucha Treatment on Small Intestinal Lesions in Indomethacin‐Administered Animals

**FIGURE 4:**
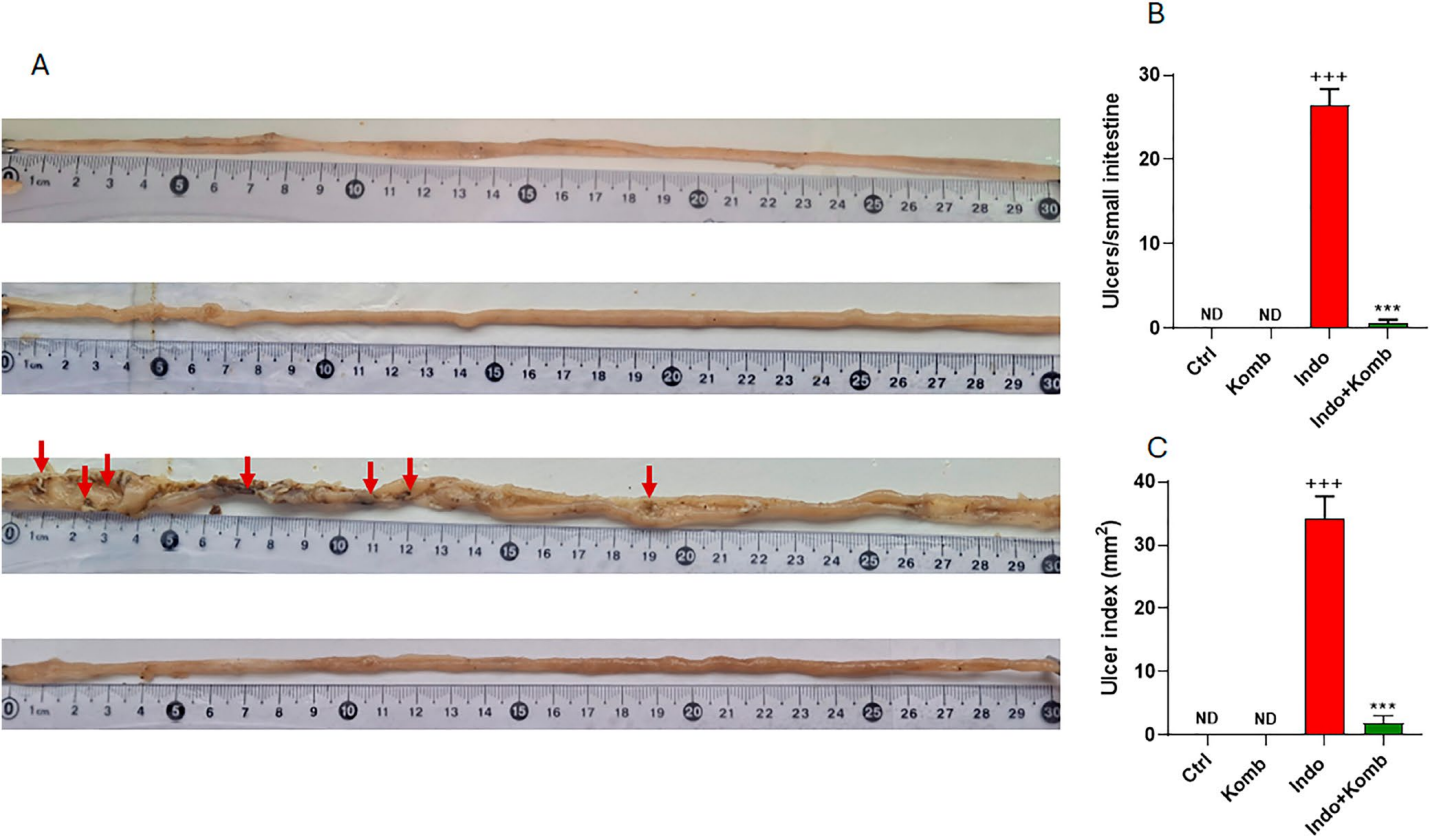
Effect of kombucha supplementation on small intestine ulceration in rats with indomethacin‐induced enteric damage. Representative images of small intestine for each experimental group (A). Red arrows indicate ulcers. Ulcer number (B) and index (C) on small intestine of rats. ^+++^ *p* < 0.001 vs. Ctrl. ***p < 0.001 vs. Indo.

The number and area of ulcers were determined on the inner side and along all small intestines (Figure 4A). As expected, no apparent ulcers were observed in Ctrl and Komb groups. The administration of indomethacin induced severe damage in the intestine, which was reflected in a significant increase in the amount of ulcers and the ulcer index in Indo animals (CI 20.95 to 31.85 ulcers and 24.63 to 43.89 mm^2^; *p* < 0.001, respectively). In contrast, with the kombucha treatment, indomethacin administration generated significantly less damage in the intestinal mucosa of animals, as the amount and the area of ulcers were dramatically reduced (CI −0.5106 to 1.711 ulcers and −1.531 to 5.272 mm^2^; *p* < 0.001 for both parameters) (Figure 4B,C).

### 3.4 Effect of Kombucha Treatment on Hematological Parameters in Indomethacin‐Administered Animals

**FIGURE 5:**
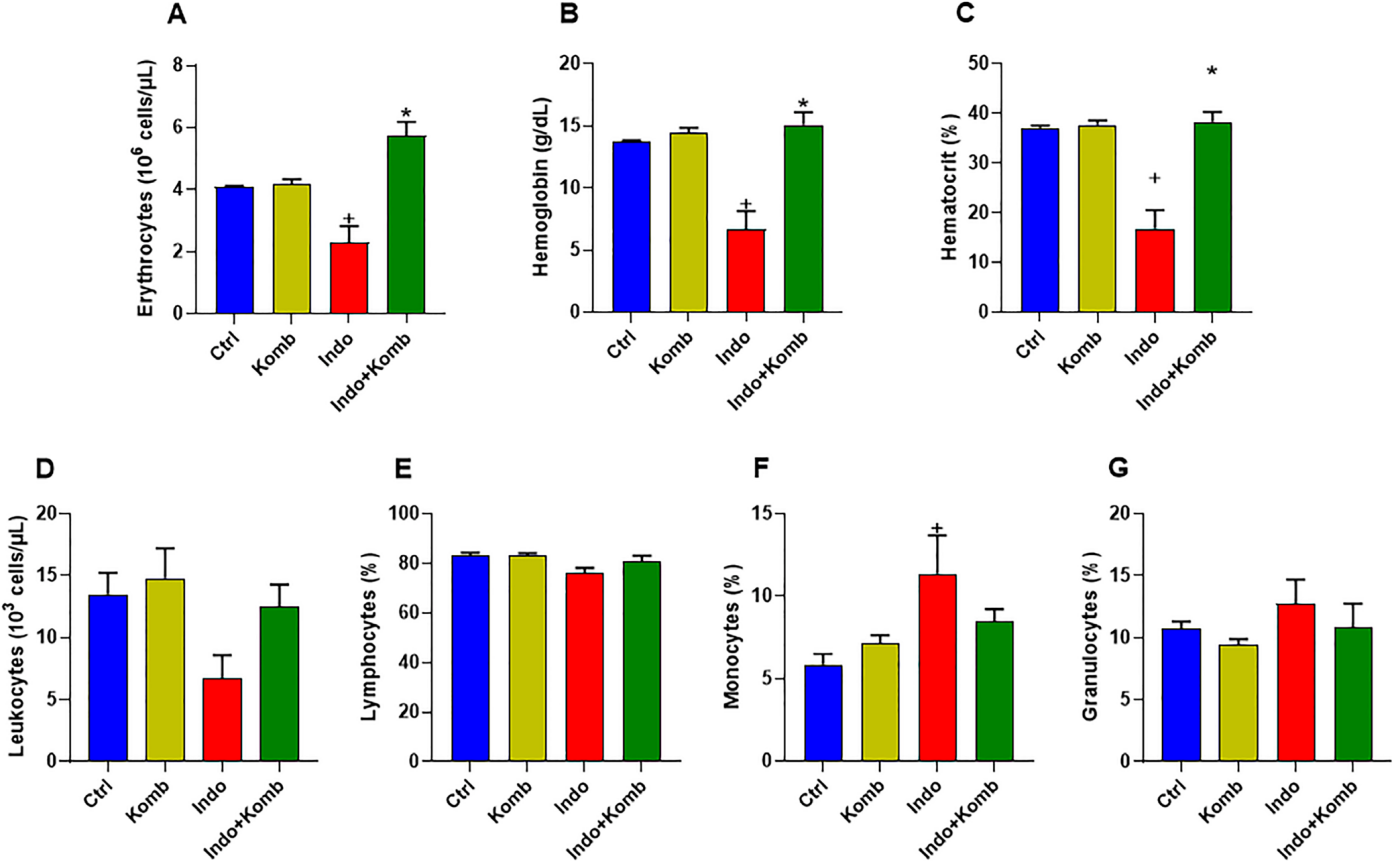
Effects of kombucha supplementation on blood parameters of indomethacin‐induced enteric damage. Erythrocyte count (A), hemoglobin amount (B), hematocrit value (C), leukocytes absolute count (D), and differential relative of lymphocytes (E), monocytes (F), and granulocytes (G). ^+^ *p* < 0.05 vs. Ctrl; *p < 0.05 vs. Indo.

There was no change in the number of erythrocytes, hemoglobin, and hematocrit levels when animals received only kombucha, since those hematological parameters were unchanged compared to the Ctrl group. Commonly, anemia is an adverse effect produced in NSAID users due to the constant mucosal bleeding (Lim et al. 2020). Accordingly, indomethacin‐induced damage animals (Indo group) had significant reductions of 43.67%, 51.24%, and 37.97% in erythrocytes number, hemoglobin concentration, and hematocrit levels (CI 0.826 to 3.770 × 10^6^ cells/μL, 2.577 to 10.78 g/dL and 6.486% to 27.15%), respectively, compared to the Ctrl group (CI 3.990 to 4.186 × 10^6^ cells/μL, 13.36 to 14.04 g/dL and 35.88% to 38.36%; *p* = 0.0115, 0.03, 0.012, respectively). These down‐regulatory effects were effectively avoided in the Indo‐Komb group, since the number of erythrocytes, hemoglobin, and hematocrit levels were 1.50‐, 1.25‐, and 1.09‐fold (4.564 to 6.940 × 10^6^ cells/μL, 12.24 to 17.92 g/dL and 32.77% to 43.87%), respectively, higher than that of the Indo group (CI 0.826 to 3.770 × 10^6^ cells/μL, 2.577 to 10.78 g/dL and 6.486% to 27.15%; *p* = 0.0201, 0.017, 0.0109), and similar to those of the Ctrl animals (Figure 5A,B).

Absolute values of leukocytes had no significant changes among experimental groups, although there was a tendency to decrease in the Indo group (CI 1.532 to 11.91 × 10^3^ cells/μL), which was prevented in the Indo‐Komb group (CI 7.445 to 17.51 × 10^3^ cells/μL). Besides, the relative values of lymphocytes and granulocytes showed no significant difference among experimental groups; however, monocyte values had a significant increase of 0.93‐fold in the Indo group (CI 4.896 to 17.83%) when compared to the Ctrl group (CI 4.068% to 7.652%; *p* = 0.0329), and a slight tendency to decrease by 25.70% in the Indo‐Komb group (CI 6.288% to 10.59%) in comparison to the Indo group (*p* = 0.5160) (Figure 5D–G).

### 3.5 Effect of Kombucha Treatment on Small Intestinal Histology in Indomethacin‐Administered Animals

**FIGURE 6:**
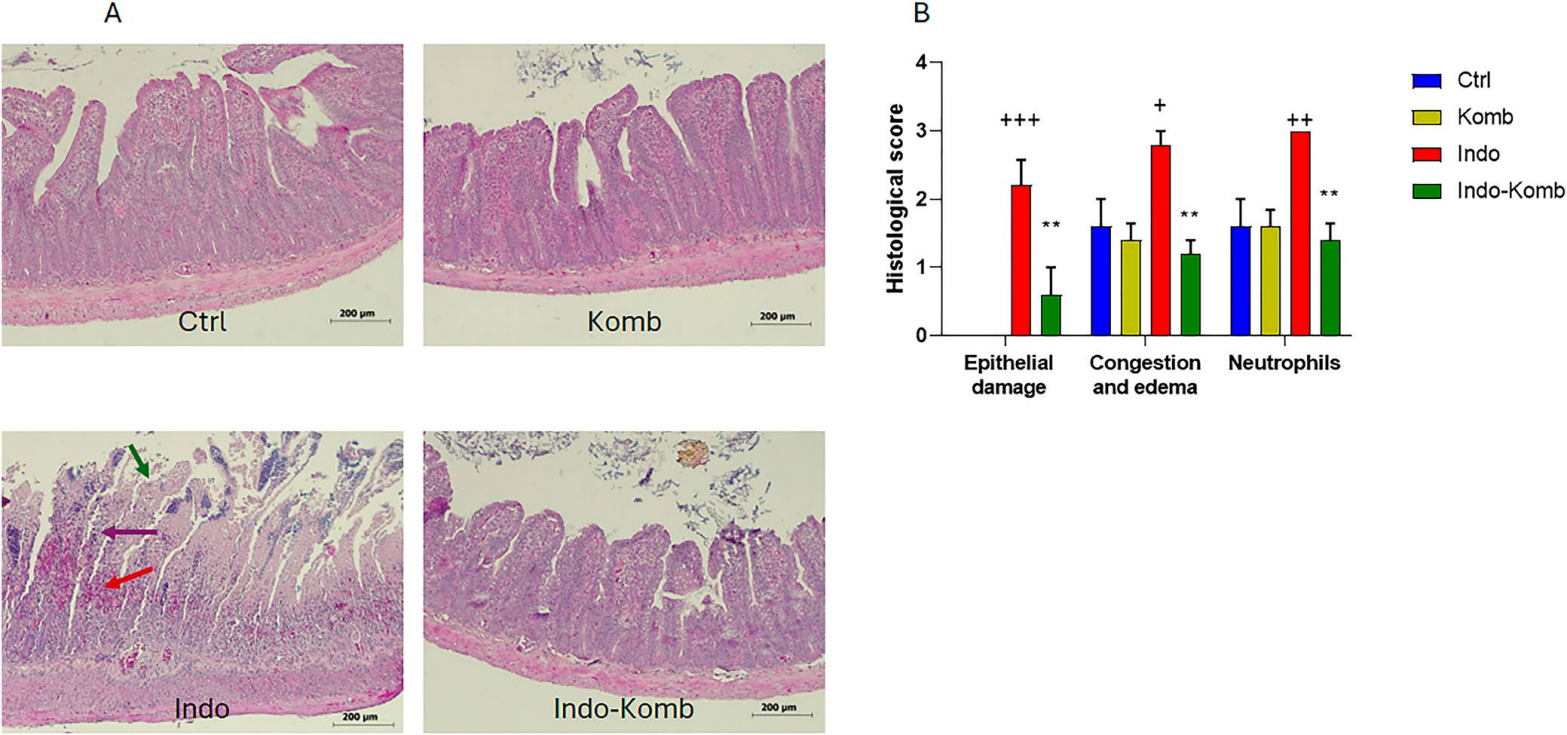
Effects of kombucha supplementation on enteric damage induced by indomethacin administration in rats. Representative images (objective 10×) of hematoxylin and eosin‐stained slides from small intestine for each experimental group. Epithelial damage (green arrow), vascular congestion (red arrow), neutrophils infiltration (purple arrow) (A). Histological scores are based on epithelia damage, congestion/edema, and neutrophil infiltration (B). ^+^ *p* < 0.05, ^++^ *p* < 0.01, and ^+++^ *p* < 0.001 vs. Ctrl. **p < 0.01, vs. Indo.

We evaluated the effect of prophylactic intake of kombucha on small intestine morphological alterations related to indomethacin daily administration. As shown in Figure 6A, indomethacin‐induced enteric damage revealed obvious small‐intestine epithelial shedding with villi fracture, and increased vascular congestion in villi with influx of inflammatory cells (Indo group). All these pathological features were not present in the small intestine of Ctrl and Komb animals. In the Indo‐Komb group, the morphology of the villus was retained despite indomethacin administration. To quantify the intestinal alterations, a histological score that encompasses epithelial damage, congestion and edema, and neutrophil infiltration was used (Lázár et al. 2019). Morphometry revealed an average 1.6‐fold higher score in intestines of the Indo animals (CI 1.162 to 3.239, 2.245 to 3.355 and 3.0, respectively) than the score of Ctrl and Komb animals (CI 0.0, 0.489 to 2.711 and 0.489 to 2.711; *p* < 0.001 for epithelial damage, *p* = 0.0297 for congestion and edema, and *p* = 0.0053 for neutrophil infiltration) (Figure 6B). Nevertheless, when animals were treated with kombucha before and during indomethacin administration, histological score was significantly lowered (CI −0.0511 to 1.711, 0.645 to 1.755 and 0.719 to 2.080, respectively) compared to animals without treatment (*p* = 0.0024 for epithelial damage, *p* = 0.0024 for congestion and edema and *p* = 0.0017 for neutrophil infiltration). Thus, according to histological examination, kombucha administration significantly protected the intestine from inflammation‐related damage.

### 3.6 Effect of Kombucha Treatment on Intestinal Barrier Function in Indomethacin‐Administered Animals

**FIGURE 7:**
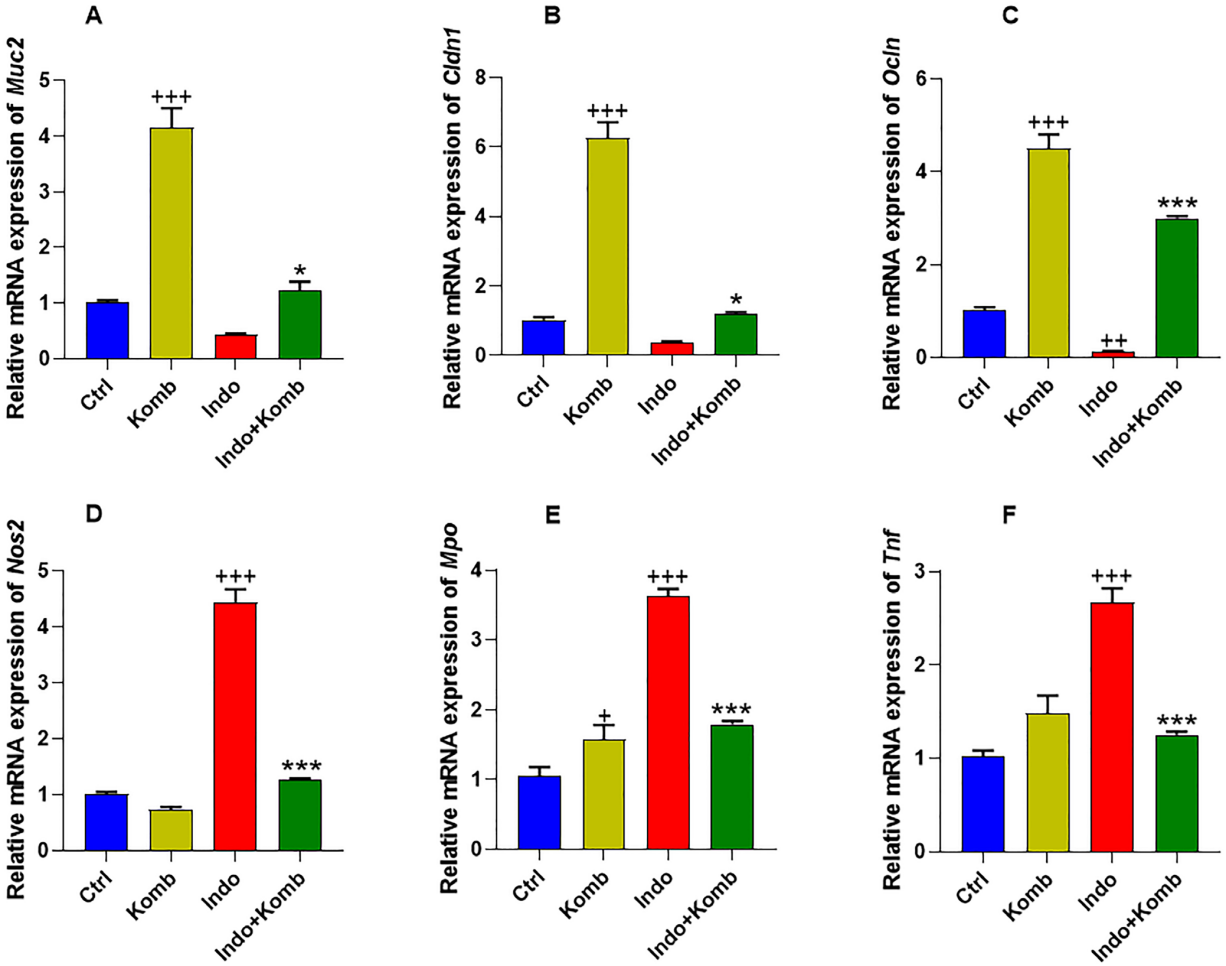
Effects of kombucha supplementation on the expression of molecular markers of intestinal barrier integrity: *Muc2* (A), *Cldn1* (B), *Ocln* (C); oxidative stress: *Nod2* (D), *Mpo* (E); and inflammation: *Tnf* (F). ^+^ *p* < 0.05, ^++^ *p* < 0.01, and ^+++^ *p* < 0.001 vs. Ctrl. *p < 0.05 and ***p < 0.001 vs. Indo.

The intestinal epithelial barrier, mainly represented by mucus secretion and tight junction proteins, is impaired when NSAIDs are administered (Carrasco‐Pozo et al. 2013; Shaik and Eid 2022; Chen et al. 2024). Therefore, we evaluated the expression of *Muc2*, *Cldn1*, and *Ocln* as markers for intestinal barrier function (Figure 7). Strikingly, oral administration of kombucha in healthy animals (Komb group) significantly increased the expression of *Muc2*, *Cldn1*, and *Ocln* by 3.12‐, 5.12‐, and 4.71‐fold (CI 3.322 to 4.984, 5.205 to 7.327 and 3.782 to 5.215, respectively) compared with the control animals (CI 0.9030 to 1.111, 0.8378 to 1.206 and 0.8608 to 1.173; *p* < 0.001 for each marker). In turn, indomethacin administration dramatically decreased by 56.45%, 62.59%, and 89.62% (CI 0.412 to 0.466, 0.325 to 0.440 and 0.112 to 0.147) the expression levels of those mRNA compared with the Ctrl group (*p* = 0.0013 for *Ocln*). The rats of the Indo‐Komb group could effectively reverse the loss of those intestinal barrier gene expressions since they were 1.77‐, 2.13‐, and 35.68‐fold higher (CI 0.819 to 1.617, 1.081 to 1.318 and 2.887 to 3.107, respectively) than that observed in the Indo group (*p* = 0.027, 0.024, < 0.001, respectively) (Figure 7A–C). Additionally, intestinal barrier dysfunction can lead to constant translocation of microbes to the submucosal layer, which then facilitates inflammation and oxidative stress (Dmytriv et al. 2024). Then, we analyzed the expression mRNA levels of *Nos2*, *Mpo*, and *Tnf* as inflammation and oxidative damage markers. In the rats with indomethacin‐induced enteric damage, the expression levels of *Nos2*, *Mpo*, and *Tnf* were found to be significantly 3.41‐, 2.44‐, and 1.62‐fold higher (CI 3.902 to 4.985, 1.051 to 2.087 and 2.313 to 3.023) than those in the Ctrl group (CI 0.9078 to 1.105, 0.7443 to 1.356 and 0.8554 to 1.177; *p* < 0.001 for each marker). Furthermore, kombucha intervention prevented this inflammatory and oxidative response, as Indo‐Komb animals significantly decreased by 71.66%, 50.48%, and 53.18% the expression of *Nos2*, *Mpo*, and *Tnf*, respectively (CI 1.186 to 1.333, 1.678 to 1.908 and 1.164 to 1.335; *p* < 0.001 for each marker) (Figure 7D–F).

### 3.7 Effect of Kombucha Treatment on Cecal Microbiota in Indomethacin‐Administered Animals

**FIGURE 8:**
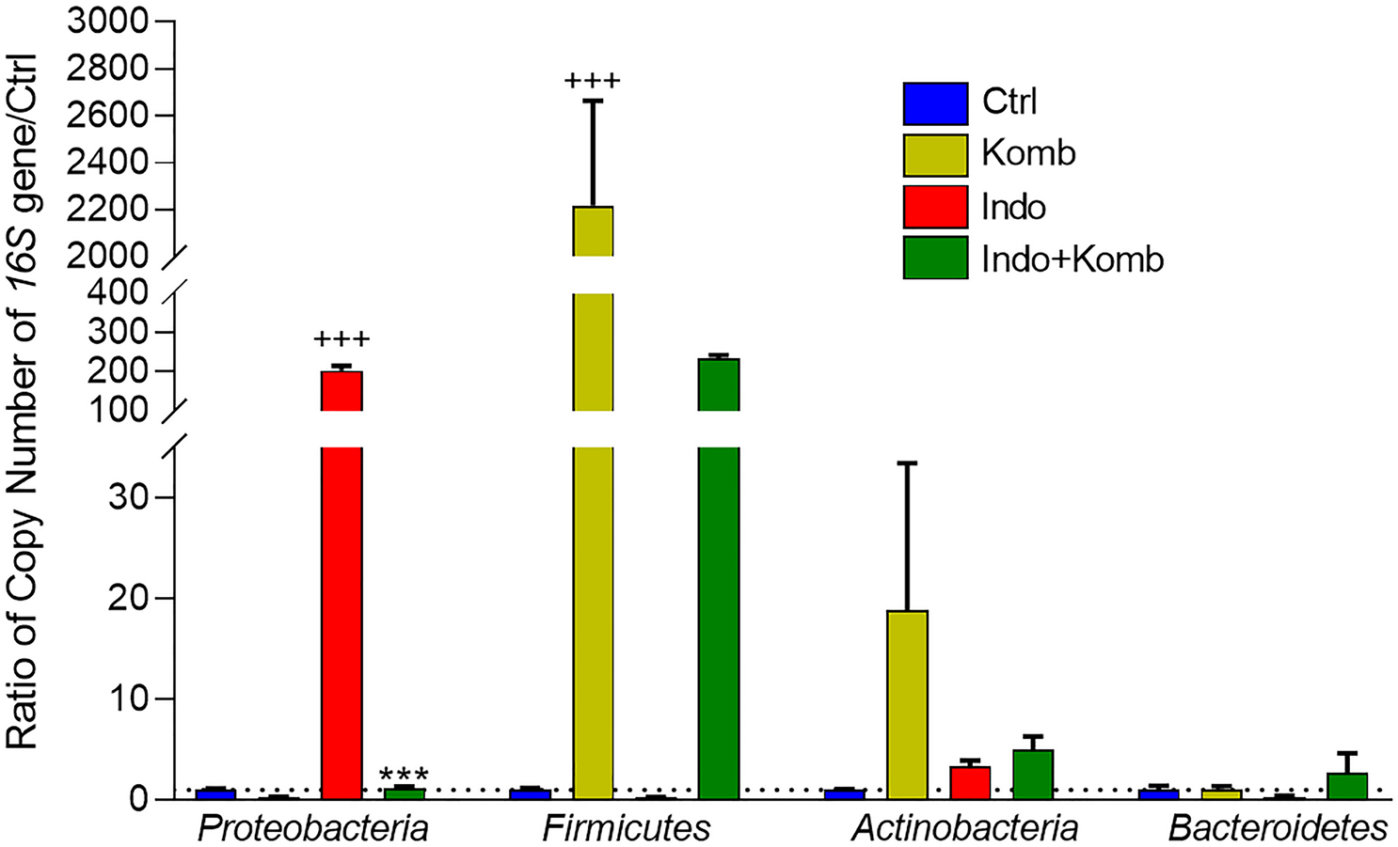
Effects of kombucha supplementation on the four main microbiota bacterial phyla: *Proteobacteria*, *Firmicutes*, *Actinobacteria*, and *Bacteroidetes*, relative to the control group. ^+++^ *p* < 0.001 vs. Ctrl; ***p < 0.001 vs. Indo.

The composition of gut microbiota may be influenced by NSAID administration. NSAID‐induced dysbiosis is responsible for worsening enteric damage since intestinal barrier function is lost and inflammation is activated (Zádori et al. 2023). To analyze the effect of kombucha supplementation in animals with indomethacin‐induced enteric damage, real‐time qPCR was performed on cecal DNA from animals of all the groups using specific primers for the *16S* rRNA gene for the dominant bacterial phyla, *Proteobacteria*, *Firmicutes*, *Bacteroidetes*, and *Actinobacteria*. As shown in Figure 8, indomethacin‐induced enteric damage was accompanied by a significant increase of *Proteobacteria* by 2.31 log‐fold (Ctrl group CI 0.728 to 1.272; Indo group CI 173.1 to 232.8; *p* < 0.001); although this effect was prevented in the Indo‐Komb group since a significant reduction by 2.24 log‐fold was observed (Indo‐Komb group CI 0.752 to 1.550; *p* < 0.001), which represents a similar level to that of the control group. It was noteworthy that kombucha intervention in healthy rats significantly reduced the *Proteobacteria* by 0.73 log‐fold (CI 0.150 to 0.380) and increased the abundance of *Firmicutes* by 3.34 log‐fold (CI 1069 to 3366) in cecal samples when compared to Ctrl animals (CI 0.622 to 1.378; *p* < 0.001). The effect of kombucha on *Firmicutes* was maintained even when indomethacin was administered, as Indo‐Komb animals showed an increase of 3.06 log‐fold (CI 214.2 to 253.6) in bacterial counts of this phylum as compared to the Indo rats (CI 0.096 to 0.311). In addition, the abundance of *Actinobacteria* and *Bacteroidetes* did not suffer any change among groups. These results indicated that kombucha modulated gut microbiota dysbiosis by promoting bacterial proliferation of *Firmicutes* and inhibiting *Proteobacteria*, which may lead to alleviation of indomethacin‐induced damage.

## 4 Discussion

NSAID‐induced enteric damage is an important challenge for public health systems due to uncontrolled access to the drug and the high demand in users with no medical supervision. High incidence of adverse events, such as ulcers, intestinal bleeding, perforations, and malabsorption of nutrients, is observed in 15%–30% of long‐term NSAID users (van de Laar et al. 2023). Under these circumstances, there is an increasing interest in strategies to alleviate intestinal damage.

The primary use for NSAIDs is to alleviate pain, fever, and inflammation. These actions are achieved through the inhibition of COX‐2, an enzyme induced during inflammation to produce mediators implicated in this process, such as PGs, thromboxanes, and leukotrienes. However, most NSAIDs also inhibit COX‐1, which is constitutively expressed in intestinal epithelial cells and has an important role in the maintenance of intestinal barrier integrity through the production of PGE2 (Gandhi et al. 2017; Peng et al. 2017; Sigthorsson et al. 2002). As an adverse consequence, during NSAID intake, there is a decrease in mucus secretion, impairment of epithelial cell regeneration, and down‐regulation of tight junctions (Bjarnason et al. 2018; Carrasco‐Pozo et al. 2013; Chen et al. 2024; Miyoshi et al. 2017; Shaik and Eid 2022; Shi et al. 2014). Additionally, NSAIDs compromise intestinal barrier function by induction of apoptosis of intestinal cells, production of reactive oxygen species, endoplasmic reticulum stress, and mitochondrial dysfunction (Boonyong et al. 2020; Carrasco‐Pozo et al. 2012). In the present study, we observed that indomethacin‐induced enteric damage can be prevented by daily kombucha supplementation, since weight loss, anemia, intestinal edema, and ulcer index were diminished in all animals, while survival rate was increased.

In recent years, there has been a growing interest in the study and application of functional foods. It has been reported that diverse natural extracts possess antioxidant activity, which may lead to reducing mitochondrial dysfunction, oxidative stress, and apoptosis in intestinal epithelial cells (Boonyong et al. 2020; Salamone et al. 2024). Supplementation with natural antioxidants has shown intestinal protective effects, which lead to alleviation of ulcers, bleeding, and inflammation (Cervantes‐García et al. 2020; Cheung et al. 2014; Ghafarzadeh et al. 2019). Considering this evidence, the prophylactic effect of kombucha supplementation on NSAID‐enteric damage might be partly mediated by its rich content of antioxidant polyphenols (Martínez Leal et al. 2018; Zhou et al. 2022), which may help to relieve oxidative stress and consequently prevent ulcer formation. In a healthy state, daily kombucha consumption favors an antioxidant state by modulating antioxidant enzymes, such as superoxide dismutase and catalase (Nogueira et al. 2025), and additionally induces an increase in the abundance of the family *Akkermansiaceae*, which may help to ameliorate future damage when an insult is presented (Costa et al. 2025).

The intestinal barrier function maintains a selective separation between internal tissues and the contents of the intestinal lumen. It is composed of mucus secretion and epithelial cells hermetically connected by tight junction proteins, such as occludin, claudin, and zonula occludens (ZO) (Arumugam et al. 2025; Dmytriv et al. 2024). Studies have shown that supplementation with natural products rich in antioxidants may improve the intestinal barrier function (Qin et al. 2024). During kombucha fermentation, a broad range of bioactive compounds with functional properties are produced, such as vitamin B9 or folate, SCFAs (mainly acetate and butyrate), polyphenol compounds, and flavonoids (de Santana Khan et al. 2024). Previous reports have shown that kombucha supplementation improves the mRNA expression of MUC2, occludin, claudin‐1, ZO‐1, and ZO‐2 in mice models of colitis and type 2 diabetes (Pakravan et al. 2019; Xu et al. 2022). This effect could be attributable, at least in part, to the SCFAs and folate, since studies suggest that there is a high association between those bacterial metabolites and intestinal barrier function (Costa et al. 2020). Although the majority of SCFAs are used as an energy source for the host, small amounts of SCFAs have biological functions, such as promoting mucosal immune cell activity and maintaining the integrity of the intestinal barrier function, thereby regulating intestinal inflammation. Modulation of inflammatory responses is mediated by SCFA receptors, such as the free fatty acid receptor (FFAR) 2, a G protein‐coupled receptor found in enterocytes, leukocytes, and other human cell types (Schlatterer et al. 2021). Acetate and propionate activate FFAR2, leading to decreased production of TNF‐α and increased IL‐10 levels (Han et al. 2024; Masui et al. 2013). In addition, butyrate and propionate influence transcription in a receptor‐independent manner by inhibiting histone deacetylases. Butyrate induces the expression of FoxP3, which enhances extrathymic T regulatory cell differentiation (Arpaia et al. 2013). Therefore, kombucha consumption delivers SCFAs that help to regulate inflammation.

Besides, folate and acetate have demonstrated promotion of epithelial cell renewal and resistance in the intestine (Deleu et al. 2023; Zhang et al. 2023). The increase in acetate production in streptozotocin‐induced type 2 diabetic rats after treatment with a mix of probiotics is associated with an improvement in the expression of tight junction genes claudin‐1, occludin, ZO‐1, and MUC2 and a decrease in the secretion of inflammatory cytokines IL‐1β, TNF‐α and IL‐6 (Manaer et al. 2025). Notably, kombucha has a high acetate content and promotes its production in type‐2 diabetic rats, which leads to increased expression of claudin‐1, occludin, ZO‐1, and MUC2, as well as a consequent reduction of TNF‐α and IL‐6 (Xu et al. 2022). Our results are in accordance with these previous insights, since indomethacin‐induced enteric damage was remarkably reduced in kombucha‐treated animals by increased mRNA expression of *Muc2*, *Cldn1*, and *Ocln*, and this improvement of the intestinal barrier integrity positively impacted the reduction of inflammatory markers *Nos2*, *Mpo*, and *Tnf*.

It is well documented that NSAIDs cause intestinal dysbiosis, which leads to worsening of the mucosal damage through mechanisms related to activation of inflammation by the proliferation of Gram‐negative bacteria and altered bile acid metabolism (Lázár et al. 2021). In this context, it has been demonstrated that probiotics consumption may alleviate NSAID‐induced damage, such as *Lacticaseibacillus casei*, *L. paracasei* , *Lactiplantibacillus plantarum, Bifidobacterium breve*, *B. longum* , *Saccharomyces boulardii* (de Vos et al. 2017; Fornai et al. 2020; Monteros et al. 2021; Mortensen et al. 2019; Simon O'Brien et al. 2023). In our experimental model using indomethacin as NSAID, intestinal damage is associated with the increase in *Proteobacteria* in cecal content, a phylum mainly represented by Gram‐negative pathogenic enterobacteria (Shealy et al. 2021), which suggests that these bacteria may contribute to intestinal inflammation. Kombucha supplementation controlled the levels of *Proteobacteria* to levels comparable to those of the control group. This effect could be attributable to the high content of polyphenols and probiotics in kombucha (Bhattacharya et al. 2016). *L. plantarum*, a probiotic bacterium present in kombucha, has inhibitory properties against pathogenic microbes (Nguyen et al. 2015). Additionally, the increased abundance of *Firmicutes* that we observed in the animals treated with kombucha emphasizes these results. Moreover, SCFA‐producing bacteria belong to the *Firmicutes* phylum (Deleu et al. 2021). Thus, the ameliorating effect on indomethacin‐induced enteric damage is associated with a beneficial prevention of dysbiosis by kombucha consumption.

The current study provides pre‐clinical proof of concept that kombucha may represent a holistic‐ and scientific‐based approach for the management of NSAID‐induced enteric damage in at‐risk populations, such as patients with rheumatoid arthritis, osteoarthritis, and other musculoskeletal disorders, particularly older adults, who are vulnerable to NSAID complications. Clinical studies may provide evidence on this subject.

Although these results support the idea that kombucha has nutraceutical properties that prevent enteric damage induced by NSAID consumption, we consider that our study presents some limitations that should be addressed, such as the use of experimental groups with male and female animals to eliminate sex‐specific variations in the responses and aged animal models. In addition, characterization of the microbial profile of diverse SCOBYs and evaluation of their effect at different doses in a model of indomethacin‐induced enteric damage may provide insights into the possible probiotics and postbiotics responsible for the protective effect. Finally, the long‐term effects of kombucha on NSAID‐induced enteric damage should be investigated to confirm its nutraceutical benefits.

In this study, we demonstrated that kombucha supplementation can prevent indomethacin‐induced enteric damage through its antioxidant, anti‐inflammatory, and intestinal microbiota‐restoring properties. These results provide strong evidence to consider kombucha, a popular fermented beverage, as a science‐based strategy in the prevention of intestinal injury due to NSAID administration.

## Author Contributions

**Atziri A. Varela‐Mendoza:** methodology (equal). **Ma. Magdalena Martínez‐Flores:** methodology (equal). **Melanie G. Paz‐Jiménez:** methodology (equal). **Fernanda García‐Acevedo:** methodology (equal). **Laura E. Córdova‐Dávalos:** data curation (supporting), methodology (supporting). **Tonatiuh Barrios‐García:** data curation (supporting), methodology (supporting). **Ma. Consolación Martínez‐Saldaña:** data curation (supporting), methodology (supporting). **Valeria Salinas‐Guardado:** methodology (supporting). **Mariela Jiménez:** data curation (supporting), formal analysis (supporting). **Eva Salinas:** conceptualization (equal), project administration (equal), supervision (equal), writing – review and editing (equal). **Daniel Cervantes‐García:** conceptualization (equal), project administration (equal), supervision (equal), writing – original draft (lead), writing – review and editing (equal).

## Ethics Statement

This study was approved by the Research Ethics Committee of the Autonomous University of Aguascalientes.

## Conflicts of Interest

The authors declare no conflicts of interest.

## Data Availability

Data available on request from the authors.
